# Maternal High-Fat Diet Induces Long-Lasting Defects in Bone Structure in Rat Offspring Through Enhanced Osteoclastogenesis

**DOI:** 10.1007/s00223-020-00801-4

**Published:** 2021-01-02

**Authors:** Priyanka Kushwaha, Seva G. Khambadkone, Mengni Li, Ethan J. Goodman, Nandini Aravindan, Ryan C. Riddle, Kellie L. K. Tamashiro

**Affiliations:** 1 Department of Orthopaedic Surgery, Johns Hopkins University School of Medicine, 720 Rutland Avenue, Ross 209, Baltimore, MD 21205 USA; 2 Department of Psychiatry and Behavioral Sciences, Johns Hopkins University School of Medicine, 720 Rutland Avenue, Ross 618, Baltimore, MD 21205 USA; 3 Cellular and Molecular Medicine Graduate Program, Johns Hopkins University School of Medicine, Baltimore, MD USA; 4 Research and Development Service, Baltimore Veterans Administration Medical Center, Baltimore, MD USA

**Keywords:** Maternal diet, Bone, Osteoclast, Developmental origins of health and disease

## Abstract

Maternal stressors during the prenatal and perinatal periods are associated with increased susceptibility for and severity of chronic disease phenotypes in adult offspring. In this study, we used a rat model of maternal high-fat diet (HFD) exposure during pregnancy and lactation to investigate the impact on skeletal homeostasis in offspring. In the distal femur, young male and female offspring (up to 3 weeks of age) from dams fed a HFD exhibited marked increases in trabecular bone volume relative to offspring from dams fed a chow diet, but this was followed by sustained bone loss. By 15 weeks of age, male offspring of HFD fed dams exhibited a 33% reduction in trabecular bone volume fraction that histomorphometric analyses revealed was due to a nearly threefold increase in the abundance of bone-resorbing osteoclasts, while there were no differences between female control and HFD offspring by 15 weeks of age. The osteoblastic differentiation of male offspring-derived bone marrow stromal cells was not affected by maternal diet. However, osteoclastic precursors isolated from the male offspring of HFD fed dams exhibited enhanced differentiation in vitro, forming larger osteoclasts with higher expression of the fusion marker DC-STAMP. This effect appears to be mediated by a cell autonomous increase in the sensitivity of precursors to RANKL. Taken together, these results suggest that maternal stressors like HFD exposure have persistent consequences for the skeletal health of offspring that may ultimately lead to a predisposition for osteopenia/osteoporosis.

## Introduction

Maintenance of bone mass and strength in the mammalian skeleton is orchestrated by the coordinated actions of osteoclasts responsible for the resorption of old or damaged bone and osteoblasts responsible for the deposition of new bone. Prior to the attainment of peak bone mass, osteoblastic activity matches or exceeds osteoclastic activity leading to net bone gains. With increasing age or the onset of menopause, bone resorption outpaces formation and leads to an increased risk for fragility fractures. The balances of these activities are known to be regulated by a myriad of local growth factors, hormones, and biophysical signals [1]. Likewise, genome wide association studies have identified genetic determinants for both peak bone mass and the propensity for the development of osteoporosis/osteopenia [2, 3].

In this study, we examined the effects of a maternal stressor on the maintenance of bone structure in offspring. The intrauterine environment is designed to support fetal development, but increasing epidemiologic and experimental evidence indicates that maternal stressors during this period or during early postnatal life can pattern susceptibility for or severity of disease in offspring [4, 5]. Referred to as the Developmental Origins of Health and Disease (DOHaD) hypothesis, maternal stressors like under- or over-nutrition, trauma, or infection are associated with increased prevalence of metabolic dysfunction, cardiovascular disease, and mental illness in progeny. Though not as well studied, maternal stressors, especially those associated with nutrition, can also influence the trajectory of bone development and strength in offspring. As an example, a large cohort study (> 50,000 mother–child pairs) indicated that maternal consumption of a calorically dense, Western-style diet was associated with an increased risk of forearm fracture between birth and 16 years of age [6]. Conversely, consumption of a healthy diet, characterized by increased fruit and vegetable consumption and limited intake of processed food, has been associated with increased bone mineral density in offspring [7].

Studies in animal models have reported similar effects of maternal nutrition on both early skeletal development and long-term maintenance of bone structure. When skeletal homeostasis was examined in the progeny of C57BL/6 mice fed a high fat diet 4 weeks prior to conception and through weaning, a generalized decrease in mineralized tissue formation was evident at gestational day 19 [8] while femoral bone mineral density was reduced at 6 months of age [9]. Interestingly, maternal caloric restriction in this same strain also produces a reduction in bone mineral content in offspring at 6 months of age [10], suggesting that both minimal levels of nutrition and nutritional content are required for optimal skeletal health. Similar effects on early skeletal development and adult bone structure were reported by Chen and colleagues [11, 12] in a rat model, wherein 12 weeks of maternal high fat diet feeding also led to a down-regulation of osteogenic transcripts and increased senescence in isolated fetal calvarial osteoblasts.

Here, we challenged pregnant rats with a high-fat diet during a more limited period of fetal and postnatal development (gestation day 2 through the weaning) and examined both short and long-term effects on skeletal metabolism in offspring. Despite early increases in bone volume, the offspring of dams fed a high-fat diet exhibited significant bone loss as they approached adulthood. We demonstrate that this effect is primarily due to an increased abundance of osteoclastic cells and an increased sensitivity of osteoclast precursors to RANKL.

## Methods

### Animals

Pregnant female Sprague–Dawley rats were purchased from Charles River (Raleigh, NC) and received on gestation day (G) 2. Dams were randomly assigned to either a high fat diet (HFD; Research Diets D12492; 60% kcal from fat, 20% kcal from protein, 20% kcal from carbohydrates; *n* = 13) or a purified, sucrose-matched control diet (Chow; Research Diets D12450J; 10% kcal from fat, 20% kcal from protein, 70% kcal from carbohydrates; *n* = 14) and individually housed in tub cages. Rats were given ad libitum access to water and maintained on a 12-h light/12-h dark cycle. Dams were maintained on their respective diets through the weaning of offspring on postnatal day (P) 21 [13, 14].

The day after birth (P1) pups were weighed and litters were culled to ten pups each (five males and five females) to standardize nutrition and maternal care across litters. To prevent litter effects from biasing our data analysis, each litter was considered *n* = 1 and only one pup per sex per litter was used for each experiment. In cases where more than one pup per sex per litter was used, the data were averaged for that particular litter and counted as *n* = 1. At 3 weeks of age (P21), pups were weaned, housed individually and maintained on the Chow diet for the duration of the study.

At 3 weeks of age, male and female rats were food-deprived overnight for 16 h with only water available. Baseline fasted blood glucose was determined via a small tail nick by a handheld glucose meter (Freestyle; TheraSense, Alameda, CA). An oral gavage of glucose (2.0 g/kg body wt, 20% glucose in sterile water solution) was then administered. Blood glucose was then determined using the glucometer at 15, 30, 60, 90, and 120 min after glucose gavage.

On P1, 3, 5 and 15 weeks of age, male and female offspring were weighed and killed by rapid decapitation. Long bone samples were collected for microCT assessment and cell culture. A separate cohort of dams (*n* = 6/group) were euthanized at weaning (P21) after a 4 h fasting period during the light cycle for analysis of maternal bone architecture. Trunk blood was collected into EDTA coated tubes, centrifuged at 4 °C, and plasma was collected and stored at − 80 °C for later measurement of plasma leptin and insulin levels by ELISA (MilliporeSigma).

### Skeletal Analysis

Femur lengths were assessed at 3, 5 and 15 weeks of age with digital calipers, while P1 lengths were measured indirectly using microCT scans. Bone architectural analysis of excised, ethanol-fixed femurs was performed using a desktop microcomputed tomographic system (Sky Scan 1275, Bruker, Belgium) in accordance with the recommendations of the American Society for Bone and Mineral Research [15]. Samples were scanned at 70 kV and 142 μA using an aluminum filter and a resolution of 9–18 μm depending on age. Scan data was reconstructed with NRecon software (Bruker, Belgium) and analyzed with CtAn Software (Bruker, Belgium). Trabecular bone architecture was assessed in a 0.5, 1, 1.5, or 2.0 mm region of interest for P1, 3, 5 and 15 week-old offspring, respectively. Cortical parameters were examined in a 500 μm region of interest centered on the femoral mid-diaphysis. Male and female offspring at each age were examined. Trabecular bone architecture in dams was assessed in a 2.0 mm region of interest.

Static and dynamic histomorphometric analyses were performed in 15-week-old male rats. Calcein (5 mg/kg) and Alizarin (30 mg/kg) were injected i.p. 8 and 3 days, respectively, prior to sacrifice. Excised femurs were fixed in ethanol and embedded in methyl methacrylate after passing through graded ethanol and xylene washes. Sections were cut with a Microm microtome and analyzed at standardized sites under the growth plate using a semiautomatic method (Osteoplan II; Kontron) in compliance with the guidelines of the nomenclature committee of the American Society for Bone and Mineral Research [16].

### Gene Expression Analyses

Total RNA was extracted from cultured cells using Trizol (Invitrogen). One microgram of pure RNA was reverse transcribed using the iScript cDNA synthesis system (Bio-rad). Two microliters of cDNA was then subjected to PCR amplification using iQ SYBR Green Supermix (Bio-rad). Primer sequences were designed using Universal Probe Library ProbeFinder software (Roche Diagnostics). Reactions were normalized to endogenous β-actin transcripts. Primer sequences can be found in Table 1.

**Table 1:** qPCR primer sequences

Actb forward	CCCGCGAGTACAACCTTCT
Actb reverse	CGTCATCCATGGCGAACT
Osteoblastic genes	
Runx2 forward	CCACAGAGCTATTAAAGTGACAGTG
Runx2 reverse	AACAAACTAGGTTTAGAGTCATCAAGC
Col1a1 forward	TCCTGGCAAGAACGGAGAT
Col1a1 reverse	CAGGAGGTCCACGCTCAC
Bglap forward	ATAGACTCCGGCGCTACCTC
Bglap reverse	CCAGGGGATCTGGGTAGG
Tnfsf11 forward	AGACACAGAAGCACTACCTGACTC
Tnfsf11 reverse	GGCCCCACAATGTGTTGTA
Tnfsf11b forward	GAGGTTTCCAGAGGACCACA
Tnfsf11b reverse	TGTCCATTCAATGATGTCCAA
Osteoclastic genes	
Tnfrsf11a forward	CTCGGGGTCTGGGAGTTC
Tnfrsf11a reverse	TGTTTCCTGTCACGTTTCCA
Dcstamp forward	TGTGTCCTCCCGCTGAATA
Dcstamp reverse	GCTTCAAAGATGGGACGATG
Acp5 forward	GACGGGAGAGATTGGTGATG
Acp5 reverse	AGTGGGAGCAGCAGGATTT
Ctsk forward	CGACTATCGAAAGAAAGGCTATG
Ctsk reverse	AAAGCCCAACAGGAACCAC
Oscar forward	CAGCCACTGGTCATCAGTTC
Oscar reverse	GAGGTTTCCCTGGGTATAGTCC

### Osteoblastic Differentiation Assays

Plastic-adherent bone marrow stromal cells were isolated from 15-week-old male rat offspring and 10^6^ cells were seed to six well tissue culture plates. Osteoblastic differentiation was induced by culturing in αMEM supplemented with 10% FBS, 1% penicillin/streptomycin, 10^−7^ M dexamethasone, 50 μg/ml ascorbic acid and 10 mM β-glycerophosphate. Media was replaced every 48 h. On day 21 of in vitro differentiation, RNA samples were collected or cultures were washed with phosphate-buffered saline, fixed with ethanol, and stained with Alizarin Red-S (40 mM solution, pH 4.1). After drying, Alizarin red stain was extracted in 10% Acetic acid, neutralized with 10% Ammonium hydroxide, and optical density was measured at 405 nm.

### Osteoclastic Differentiation Assays

Bone marrow cells were isolated from the long bones of 15-week-old male rat offspring and cultured in α-MEM with 10% FBS, 1% penicillin/streptomycin and 50 ng/ml M-CSF for 16–18 h to separate adherent bone marrow stromal cells and non-adherent hematopoietic cells. The non-adherent population was collected by centrifugation and then cultured in 12 well plates in the presence of M-CSF and RANKL (0.5–50 ng/ml) for 5 days. Cultures were used for RNA isolation or fixed and stained using the TRAP-Leukocyte staining kit (Sigma). Multinucleated (> 3 nuclei) TRAP positive cells were counted using ImageJ software with 2–4 fields analyzed from each well.

### Statistical Analyses

Comparison between groups was performed with student *t* test using Prism software (GraphPad). In all figures, data are expressed as mean ± SEM. A *p*-value less than 0.05 was considered significant.

## Results

### HFD Feeding During Gestation and Lactation Reduces Maternal Bone Volume

**Fig. 1:**
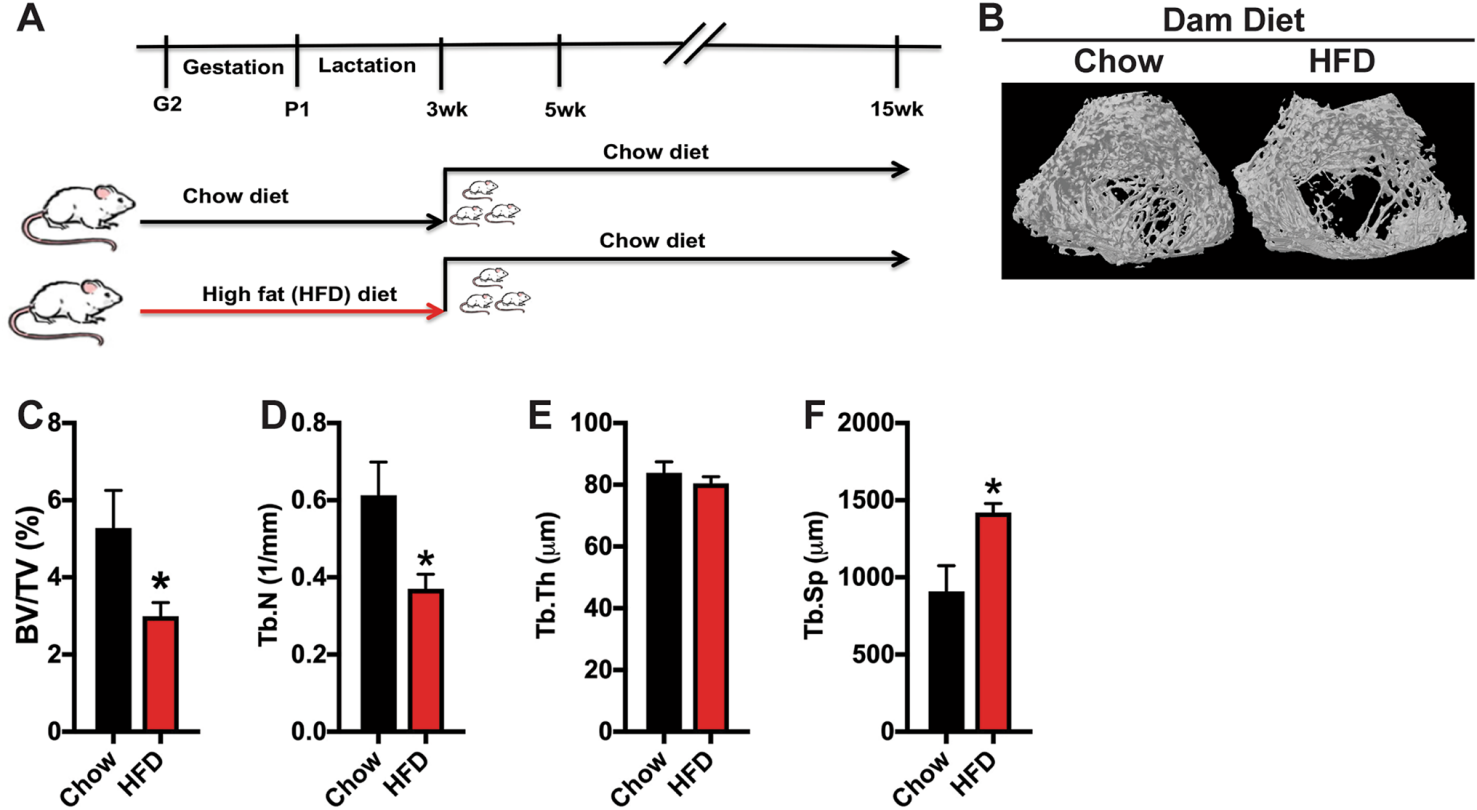
High fat diet feeding during gestation and lactation exacerbates maternal bone loss. **a** Schematic representation of experimental strategy. Pregnant rats were fed a Chow or high fat diet (HFD) from G2 through weaning at 3 weeks of age. Offspring were weaned on to a Chow diet. **b** Representative 3D reconstruction of microCT examination of trabecular bone structure in the distal femur of Chow and HFD dams. **c**–**f** Quantification of trabecular bone volume/tissue volume (**c**, BV/TV), trabecular number (**d**, Tb.N), trabecular thickness (**e**, Tb.Th) and trabecular separation (**f**, Tb.Sp) in the distal femur (*n* = 6 rats/group). All results are expressed as mean ± SEM, *p < 0.05

Maternal stressors like obesity or over-nutrition can produce long-lasting effects in offspring that increase susceptibility for or severity of disease phenotypes [4]. To examine long term effects of maternal diet on skeletal health in offspring, we fed pregnant dams Chow (10% kcal from fat) or HFD (60% kcal from fat) from day 2 of gestation (G2) through weaning at 3 weeks of age (P21) [13, 14]. Dams fed the HFD exhibited reduced food intake in grams but the total caloric intake for the HFD group was significantly greater than the Chow group due to the increased caloric density of the HFD (Table 2). Fat intake and carbohydrate intake differed according to the make-up of the diet, but caloric intake from protein was well matched. Offspring were weaned and maintained on Chow diet through the conclusion of the study (Fig. 1a).

**Table 2:** Dams body weight, food intake and plasma hormone levels

Parameter^‡^	Dam diet	
	Chow (*n* = 6)	High fat (*n* = 6)
Body weight, P21 (g)	301.3 ± 11.5	307.8 ± 11.4
Food Intake (g), gestation + lactation	2201.3 ± 73.6	1750.9 ± 62.3*
Caloric intake (kcal), gestation + lactation	7374.3 ± 246.6	9174.7 ± 326.4*
Plasma leptin, P21 (ng/ml)	3.4 ± 0.7	10.3 ± 1.8*
Plasma insulin, P21 fasted (ng/ml)	1.9 ± 0.3	1.8 ± 0.3

^‡^Values are shown as mean ± SEM

*p < 0.05

Before examining the phenotype of offspring, we examined skeletal architecture in dams at weaning. Consistent with our previous reports [17–19], there was no significant difference in body weight between dams fed a Chow or HFD on P21, but HFD dams had significantly greater plasma leptin levels with no difference in fasted insulin levels (Table 2). MicroCT analysis of the trabecular bone compartment of the distal femur revealed a more significant deterioration of trabecular structure in dams fed HFD (Fig. 1b). Relative to Chow fed dams, HFD dams exhibited a 44% reduction in trabecular bone volume with corresponding reductions in trabecular number and increases in trabecular spacing (Fig. 1c–f). Thus, HFD feeding appears to exacerbate maternal bone loss during pregnancy and lactation.

### Maternal HFD Feeding Alters Body Weight, Femur Length, and Glucose Homeostasis in Offspring

**Fig. 2:**
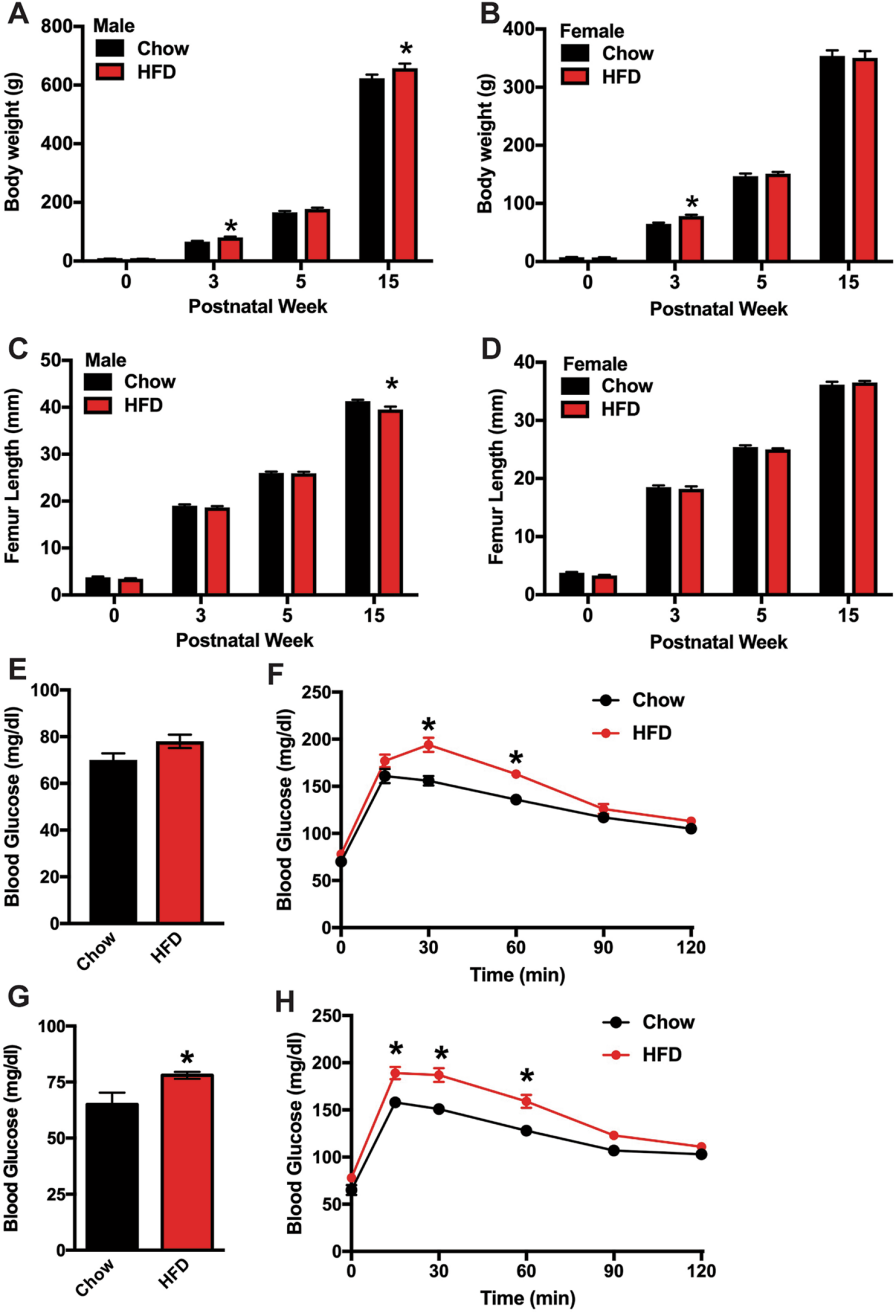
Effect of maternal diet on body weight and femur length in offspring. Body weight of male (**a**) and female (**b**) offspring were assessed on P1 (postnatal week 0) and at the indicated timepoints through 15 weeks of age (*n* = 8–15 rats/group). Femur length of male (**c**) and female (**d**) offspring were assessed at each timepoint (*n* = 8–15 rats/group). Glucose homeostasis was assessed by measuring fasting blood glucose (**e**, **g**) and performing oral glucose tolerance testing (**f**, **h** in male (**e**–**f**) and female (**g**, **h**) offspring at 3 weeks of age (*n* = 8 rats/group). All results are expressed as mean ± SEM, *p < 0.05

As a first step in determining the effect of maternal diet on offspring, we measured body weight and femur length from birth (P1) to 15 weeks of age. At P1, body weight in the offspring of dams fed a Chow and HFD were similar (Fig. 2a, b). However, by 3 weeks of age, both male and female offspring of HFD dams exhibited a 20% increase in body weight relative to offspring of Chow dams. Body weight normalized by 5 weeks of age, but male offspring of HFD dams developed an increase in body weight again by 15 weeks of age. These data are consistent with our previous findings [13].

Femur length in offspring of HFD dams were comparable to those of Chow dams (Fig. 2c, d) during the period of most rapid longitudinal growth (0–5 weeks of age, [20]). By skeletal maturity, male offspring of HFD dams exhibited a 5% decrease in femur length relative to offspring of Chow dams (Fig. 2c). Though not directly examined here, previous studies indicate that longitudinal growth between 5 weeks of age and maturity in rats is primarily due to changes in the rate of chondrocyte proliferation and alterations in cell volume [20, 21].

Assessment of fasting blood glucose levels and the performance of oral glucose tolerance testing at 3 weeks of age revealed that maternal HFD at least temporarily impaired glucose homeostasis in offspring. Fasting blood glucose levels were significantly increased in the female offspring of HFD dams (Fig. 2g), while a strong upward trend was evident in their male littermates (*p* = 0.07, Fig. 2e). Both male and female offspring of HFD dams exhibited impairments in glucose disposal during glucose tolerance testing as blood glucose levels peaked higher than the offspring of Chow dams after oral glucose administration (Fig. 2f, h). Our previous studies indicate the impairment in glucose tolerance recovers with age due to an increase in glucose-stimulated insulin secretion [17–19]. Taken together these data indicate that maternal high fat diet feeding during gestation and the lactation periods exerts long term effects on metabolism and longitudinal growth, especially in male offspring.

### Maternal HFD Feeding Induces Age-Related Defects in Trabecular Bone Volume in Offspring

**Fig. 3:**
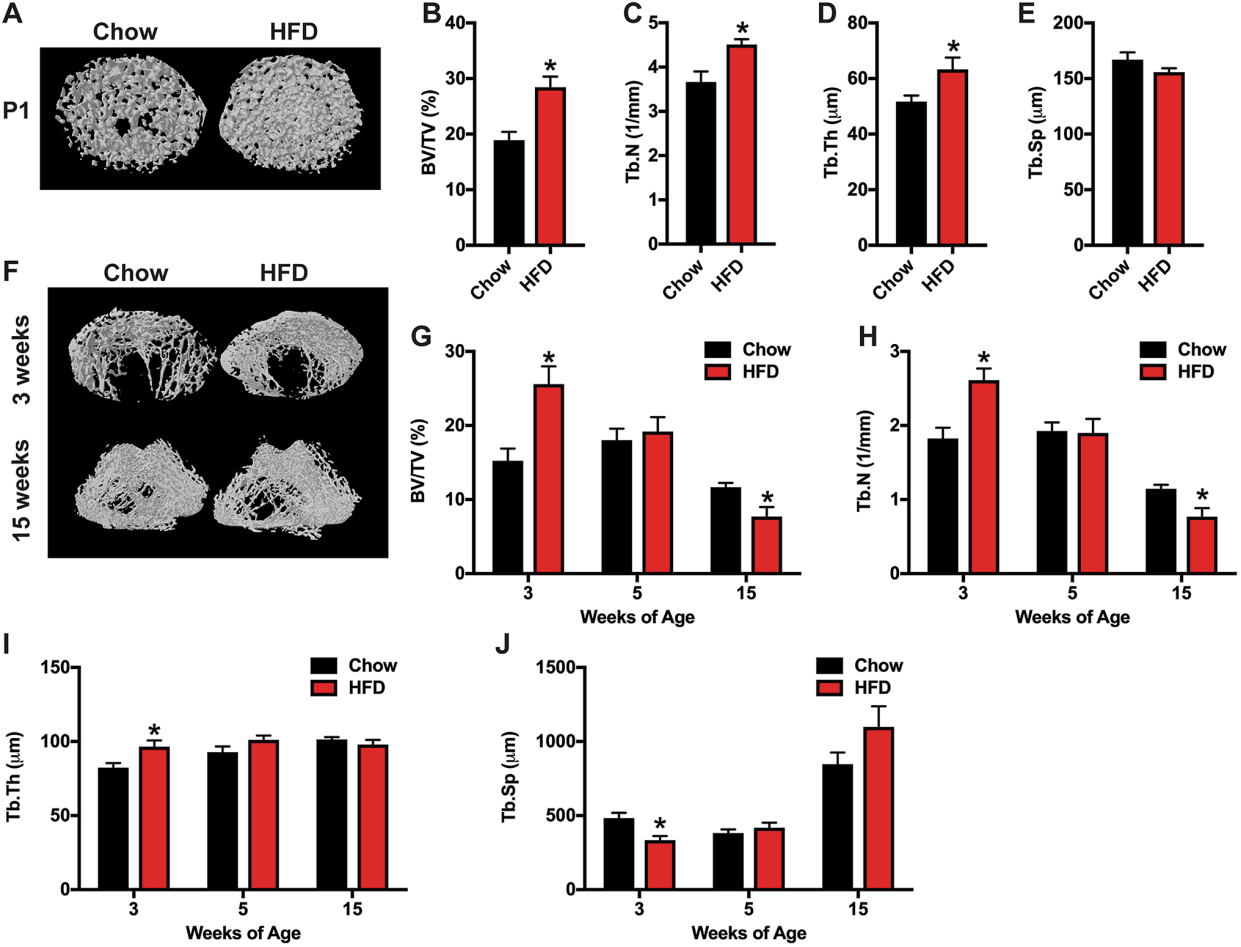
MicroCT analysis of trabecular bone in male offspring. **a** Representative microCT images of the distal femur at postnatal week 0 (P1) of the male offspring of dams fed a Chow or HFD. **b**–**e** Quantification of trabecular bone volume/tissue volume (**b**, BV/TV), trabecular number (**c**, Tb.N), trabecular thickness (**d**, Tb.Th) and trabecular separation (**e**, Tb.Sp) in the distal femur (*n* = 8–15 rats/group) at postnatal week 0. **f** Representative microCT images of the distal femur at postnatal week 3 and 15 of the male offspring of dams fed a Chow or HFD. **g**–**j** Quantification of trabecular bone volume/tissue volume (**g**, BV/TV), trabecular number (**h**, Tb.N), trabecular thickness (**i**, Tb.Th) and trabecular separation (**j**, Tb.Sp) in the distal femur (*n* = 8–15 rats/group) at postnatal weeks 3, 5 and 15. All results are expressed as mean ± SEM, *p < 0.05

We next utilized microCT analyses to examined the effects of maternal nutrition on skeletal homeostasis. In male neonates (P1), offspring from HFD dams exhibited a robust increase in femoral trabecular volume (Fig. 3a–e), secondary to increases in trabecular number and trabecular thickness, relative to offspring from Chow dams. The increase in bone volume persisted through 3 weeks of age (Fig. 3f–j), but was followed by a more rapid rate of bone loss than that evident in offspring of Chow dams. By 15 weeks of age, trabecular bone volume in offspring of HFD dams was significantly reduced (~ 33%) relative to that of offspring from Chow dams. No difference in cortical bone parameters were evident at any of these timepoints.

**Fig. 4:**
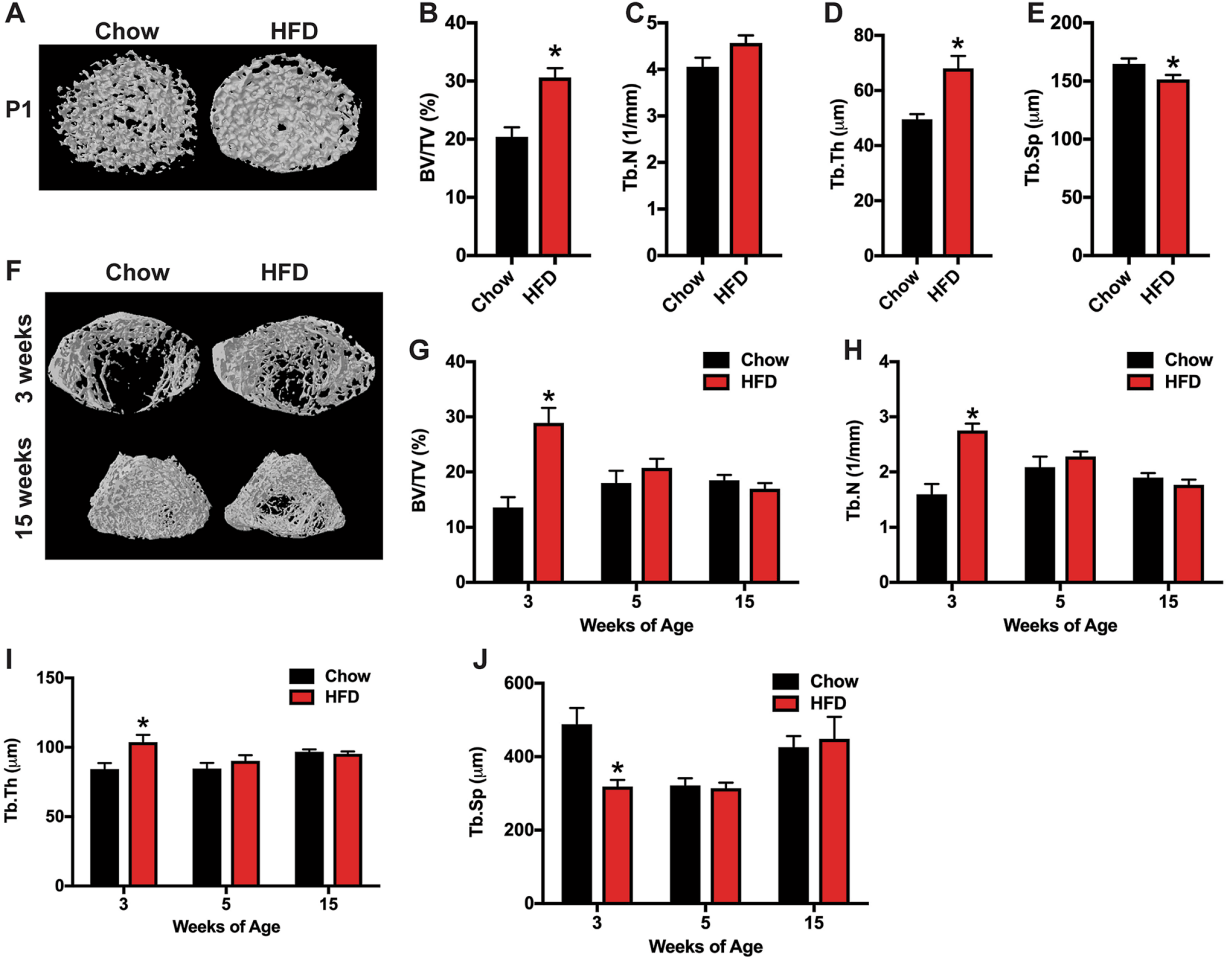
MicroCT analysis of trabecular bone in female offspring. **a** Representative microCT images of the distal femur at postnatal week 0 (P1) of the female offspring of dams fed a Chow or HFD. **b**–**e** Quantification of trabecular bone volume/tissue volume (**b**, BV/TV), trabecular number (**c**, Tb.N), trabecular thickness (**d**, Tb.Th) and trabecular separation (**e**, Tb.Sp) in the distal femur (*n* = 8–15 rats/group) at postnatal week 0. **f** Representative microCT images of the distal femur at postnatal week 3 and 15 of the female offspring of dams fed a Chow or HFD. **g**–**j** Quantification of trabecular bone volume/tissue volume (**g**, BV/TV), trabecular number (**h**, Tb.N), trabecular thickness (**i**, Tb.Th) and trabecular separation (**j**, Tb.Sp) in the distal femur (*n* = 8–14 rats/group) at postnatal weeks 3, 5 and 15. All results are expressed as mean ± SEM, *p < 0.05

Female neonates (P1) from HFD dams exhibited similarly robust increases in femoral trabecular bone volume, primarily due to an increase in trabecular thickness, when compared to offspring of Chow dams (Fig. 4a–e). As with male offspring, the increase in trabecular bone volume remained evident at 3 weeks of age in female offspring of HFD dams (Fig. 4f–j), but at the later timepoints (5 and 15 weeks) trabecular bone volume was equivalent in the two groups. These data indicated that female offspring of HFD dams also exhibit a more rapid rate of bone loss, but the effect is limited to the period between 3 and 5 weeks of age. Again, no difference in cortical bone parameters were evident at any timepoint in female offspring.

To elucidate the cellular basis for the more dramatic trabecular bone loss in offspring of HFD dams, we performed static and dynamic histomorphometric analyses in 15-week-old offspring. We focused on male offspring since this sex exhibited the most severe phenotype. Parameters of bone formation in offspring of HFD dams were not significantly different from the offspring of Chow dams, though modest increases in osteoid volume per bone volume, osteoid surface per bone surface, osteoblast numbers per bone perimeter, and mineralizing surface per bone surface were evident (Table 3). Rather, offspring of HFD dams exhibited marked increases in parameters of bone resorption. Most notably, osteoclast number per bone perimeter was nearly 3 times higher in offspring of HFD dams when compared to those from Chow dams. Therefore, maternal HFD feeding during gestation and lactation induces a state of heightened bone turnover weighted toward resorption that results in significant deficiencies in bone structure as progeny approach adulthood.

**Table 3:** Static and dynamic histomorphometry

Bone parameter^‡^	Dam diet	
	Chow (*n* = 7)	High fat (*n* = 6)
Bone formation		
Osteoid volume/bone volume (OV/BV; %)	0.22 ± 0.04	0.41 ± 0.09
Osteoid surface/bone surface (OS/BS; %)	2.88 ± 0.45	5.25 ± 1.37
Osteoid thickness (O.Th; μm)	2.06 ± 0.30	2.19 ± 0.24
Osteoblast surface/bone surface (Ob.S/BS; %)	2.27 ± 0.46	3.87 ± 0.76
Osteoblast number/bone perimeter (NOb/BPm; no./100 mm)	170.13 ± 37.70	285.91 ± 58.62
Bone erosion		
Erosion surface/bone surface (ES/BS; %)	1.51 ± 0.34	3.65 ± 0.77*
Osteoclast surface (Oc.S/BS; %)	1.40 ± 0.75	3.54 ± 0.89*
Osteoclast number/bone perimeter (NOc/BPm; no./100 mm)	36.13 ± 9.03	101.59 ± 27.61*
Bone dynamics		
Mineral apposition rate (MAR; μm/day)	1.29 ± 0.11	1.49 ± 0.13
Mineralizing surface/bone surface (MS/BS; %)	16.54 ± 1.43	21.07 ± 2.59
Bone formation rate/bone surface (BFR/BS; mm^3^/cm^2^/year)	7.98 ± 1.32	12.02 ± 2.32
Mineralization lag time (Mlt; day)	0.32 ± 0.06	0.41 ± 0.13
Osteoid maturation time (Omt; day)	1.61 ± 0.20	1.56 ± 0.23

^‡^Values are shown as mean ± SEM

*p < 0.05

### Maternal HFD Feeding Enhances RANKL Sensitivity in Bone Marrow Macrophages of Male Offspring

**Fig. 5:**
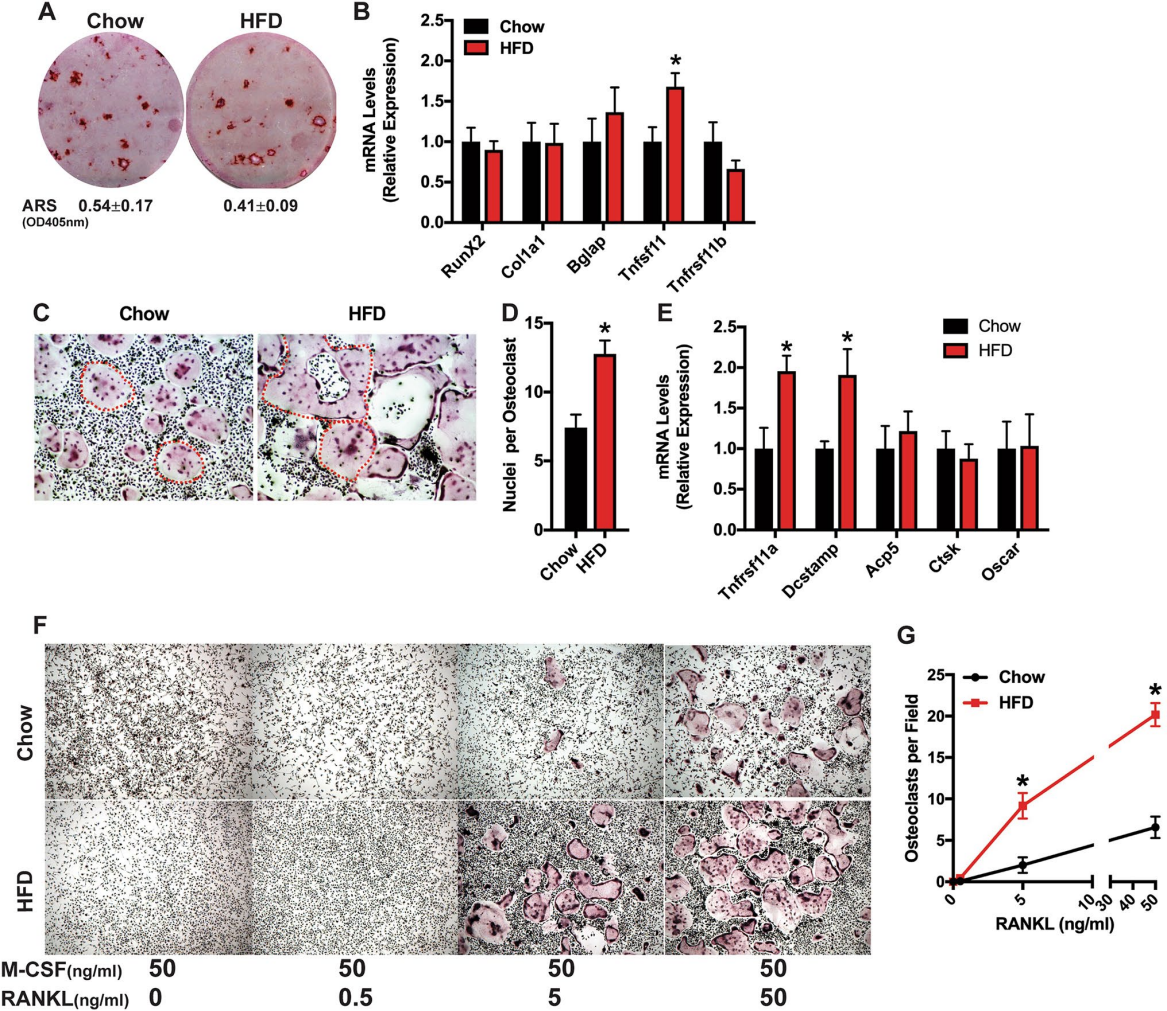
Maternal high fat diet enhances in vitro osteoclastogenesis in offspring. **a**, **b** Bone marrow stromal cells isolated from the offspring of dams fed a Chow or HFD were differentiated under osteogenic conditions for 21 days. Alizarin red staining (**a**) and qPCR (**b**) were used to assess differentiation. Quantification of Alizarin red staining after extraction is shown under the images. **c–e** Osteoclast precursors isolated from the offspring of dams fed a Chow or HFD were differentiated in the presence of M-CSF (50 ng/ml) and RANKL (50 ng/ml) for 5 days. TRAP staining with two osteoclasts per imaged outlined in red (**c**, × 6.4 original magnification), assessment of nuclei per osteoclast (**d**) and qPCR (**e**) were used to assess differentiation. **f**, **g** Osteoclast precursors isolated from the offspring of dams fed a Chow or HFD were differentiated in the presence of M-CSF (50 ng/ml) and the indicated concentration of RANKL for 5 days. TRAP staining (**f**, × 4 original magnification) and quantification of osteoclasts per field were used to assess differentiation. All results are expressed as mean ± SEM, *p < 0.05. In vitro studies were replicated in cell cultures isolated from 4 to 7 rats

To complement the histomorphometric studies, we examined the in vitro osteoblastic and osteoclastic differentiation potential of bone marrow-derived cells. Consistent with the in vivo bone formation parameters (Table 3), bone marrow stromal cell cultures isolated from male offspring of Chow and HFD dams exhibited similar capacity for mineralized nodule formation (Fig. 5a) and expression of *Runx2*, *Col1a1*, and *Bglap* (Fig. 5b) after 21 days of osteogenic differentiation. Interestingly, the expression of *Tnfsf11*, encoding RANKL, was significantly increased in stromal cell cultures from offspring of HFD dams, while the expression of *Tnfsf11b*, encoding the soluble inhibitor Osteoprotegerin, was not affected. Thus, maternal HFD feeding appears to lead to a cell autonomous increase in osteoblastic signaling to stimulate osteoclastic bone resorption.

Marked differences in osteoclastic differentiation after M-CSF and RANKL stimulation were evident in cultures of osteoclast precursors isolated from the offspring of Chow and HFD dams. TRAP staining on day 5 of in vitro differentiation revealed that cells isolated from offspring of HFD dams formed larger osteoclasts with more numerous nuclei than those isolated from the offspring of Chow dams (Fig. 5c, d). Consistent with this finding, the expression of *Tnfrs11a*, encoding RANK, and *Dcstamp*, a mediator of osteoclastic cell fusion, were significantly increased in cells cultures isolated from the offspring of HFD dams when compared to those from the offspring of Chow dams (Fig. 5e). The increase in RANK expression led us to speculate that maternal HFD feeding led to an increase in the sensitivity of osteoclastic precursors to RANKL. To test this, we performed a dose–response study wherein osteoclast precursors from the adult male offspring of Chow or HFD dams were treated with M-CSF (50 ng/ml) and 0, 0.5, 5, or 50 ng/ml RANKL for 5 days. No osteoclasts were evident in the 0 or 0.5 ng/ml RANKL treatment groups (Fig. 5f). At 5 ng/ml RANKL, robust differentiation of TRAP + osteoclasts was evident in precursors isolated from the offspring of HFD dams while few multi-nucleated TRAP + cells were evident in cultures from offspring of Chow dams (Fig. 5f, g). Altogether, these data suggest that maternal HFD leads to a sensitization of osteoclast precursors to RANKL as well as an increase in osteoblast RANKL production that in turn leads to a state of accelerated age-related bone loss in male offspring.

## Discussion

It has become clear that adverse maternal exposures during prenatal and early postnatal life can impair offspring health across the lifespan and across physiological systems. With the dramatic rise in worldwide obesity rates and globalization of the high-fat Western-style diet model, recent attention has turned to the consequences of maternal exposure to HFD and overnutrition on offspring [4]. Maternal overnutrition has been associated with increased risk for a range of adverse health effects in offspring [22], and emerging clinical and preclinical evidence suggests it may also exert long term consequences on skeletal health [6, 8, 9, 11, 12]. In this study, we used an established model of maternal HFD feeding [13, 14, 23] to examine both short and long term effects on the skeletal health of offspring. Using the same paradigm [17–19], we previously reported that both male and female HFD offspring are similar in body weight at birth, but have increased body weight by P7 and have greater adiposity by P10. Glucose tolerance is impaired in HFD offspring by 3 weeks of age, the earliest age tested, and HFD offspring are hyperleptinemic throughout the early postnatal period, but are leptin resistant and have deficits in hypothalamic leptin signaling when tested at P10 and 3 weeks of age [17–19].

In young offspring (through 3 weeks of age), maternal HFD feeding resulted in a marked increase in trabecular bone volume. Though not directly examined, this phenotype is almost assuredly due to an increase in osteoblast activity, since bone modeling is most active during this period of rapid growth [24–26]. We speculate that this early effect is due to nutritional effects as our previous studies indicated that dams fed a HFD spent more time nursing that those fed a chow diet and the offspring of HFD dams consume more milk than those of chow dams [23]. The milk of HFD fed dams also tended to have a higher concentration of fat and protein than that of chow fed dams resulting in greater energy content [23]. Although maternal bone remodeling is normally greater during pregnancy and lactation due to increased calcium requirements by the developing fetus and neonate, maternal bone loss appears to have been exacerbated in HFD dams and this could be a consequence of greater demand for milk by HFD offspring.

After 3 weeks of age, the male and to a lesser extent female offspring of HFD fed dams exhibited greater bone loss than the offspring of chow fed dams. The cellular basis for this phenotype in our study differs from previous studies that used both mice and rats and demonstrated maternal HFD feeding negatively impacted osteoblast performance in offspring [11, 12, 27]. As an example, data from the Shankar laboratory identified an increase in the senescence associated secretory phenotype in fetal calvarial osteoblasts isolated from the progeny of HFD fed rat dams [12]. In our study, we only observed a change in the abundance of osteoclasts in vivo and enhanced osteoclast differentiation in vitro. In all likelihood, the trends towards increases in osteoid parameters and the mineralizing surface are due to the coupling of osteoblast activity to heightened osteoclastic activity. The main difference between our study and the others is the length of maternal challenge with a HFD. In previous studies [11, 12, 27], dams were fed a HFD for 4 to 12 weeks prior to breeding and then through the weaning of pups. Here, dams were only challenged with HFD from gestational day 2 through weaning. These differences suggest the length of exposure to nutritional stressors dictates which cell populations involved in bone remodeling are affected.

The length of maternal exposure to HFD is likely to result in differences in the development of metabolic disturbances in dams that may then affect offspring. Our previous studies showed that HFD exposure from gestational day 2 to weaning results in changes to several metabolic indices in dams. Increases in body weight relative to Chow-fed dams is not consistently observed in HFD fed dams, but adipose tissue mass is increased by birth and coupled with the development of hyperleptinemia by G10 when compared to Chow-fed dams [14, 18, 19]. In addition, HFD fed dams exhibit impaired glucose tolerance and decreased plasma adiponectin as early as G14 [14]. We did not make direct measurements of dam metabolism during gestation or lactation here as blood collection or glucose tolerance testing would introduce additional stressors as confounding variables, but HFD dams had higher plasma leptin levels at weaning suggesting that they had greater adiposity. Longer periods of maternal HFD exposure than employed here are likely to result in more severe metabolic changes. For example, beginning maternal HFD access several weeks prior to conception, as conducted in the studies described above, results in maternal obesity and metabolic derangement [28, 29]. In addition, preconception obesity may also alter oocyte metabolism, morphology and maturation [30, 31]. Such differences in model design—preconception obesity versus perinatal HFD exposure—could thus alter the maternal metabolic milieu and subsequent severity and trajectory of the developmental environment experienced by the offspring. Nevertheless, the consistent theme regardless of the model used is that maternal diet has a significant and persistent consequence on offspring’s bone biology and health, even if the offspring themselves did not consume the HFD. In fact, weaning on to a HFD or provision of HFD to adult offspring would likely exacerbate bone remodeling and accelerate disease, as has been reported for other phenotypes affected by maternal HFD [17, 32, 33].

It is not surprising that the ultimate offspring phenotype is dependent on the timing of maternal dietary manipulation, whether the dam’s exposure occurs throughout life prior to pregnancy or only during the immediate perinatal period after fertilization has occurred. In each case the “insult” occurs at different developmentally sensitive time points for the offspring whether it is as a germ cell, early embryo, fetus, or neonate. Such outcomes from different models provide clues that may be leveraged to determine how maternal diet or metabolic state affects different stages of bone development (i.e. differentiation of precursors, longitudinal growth, coupling of formation to resorption, etc.) depending on the specific exposure windows.

Determining the mechanism by which maternal HFD feeding results in increased osteoclastogenesis will require additional study. This effect could be secondary to alterations in metabolic hormones like leptin and insulin that influence bone remodeling, and/or changes in the activity of osteocytes, which transgenic mouse models indicate are the dominant source of RANKL production in vivo [34, 35] and trigger targeted resorption in response to apoptotic signals [36]. Consistent with this later possibility, our in vitro data suggests increases in bone resorption are at least partially due to cell autonomous effects on osteoblasts and osteoclasts. Osteoblasts differentiated from bone marrow stromal cells isolated from the offspring of HFD fed dams exhibited an increase in RANKL expression even though they did not exhibit an impairment in markers of differentiation potential. These data suggest osteoblastic signaling to osteoclasts is increased. On the other hand, osteoclasts exhibited an increase in RANK expression and precursors exhibited an increase in sensitivity to RANKL. This effect could be mediated by epigenetic changes that influence the machinery that confers sensitivity to differentiation inducing signals. There is a growing appreciation of epigenetic control both in normal bone biology and in the onset and progression of musculoskeletal disease [37–39]. Epigenetic profiles that ultimately influence gene, cell, tissue and organ function are established during the perinatal period. Recent findings reveal the role of DNA methylation in control of Wnt, RANK/RANKL, and other key signaling pathways, as well as epigenetic regulation of osteoblast and osteoclast differentiation [39]. Thus, maternal diet could alter epigenetic histone modifications, DNA methylation, and non-coding RNAs in offspring to confer susceptibility to pathologic bone remodeling that persists throughout life.

In summary, our study indicates that a maternal dietary insult is sufficient to produce long term defects in skeletal remodeling that may ultimately lead to a predisposition for the development of osteopenia/osteoporosis. Future work will be directed toward understanding the cell autonomous mechanism by which maternal HFD instructs an increase in osteoclast development as well as the exploration of therapeutic interventions that offset the detrimental effects of maternal stressors.
